# Homeless Shelter Characteristics and Prevalence of SARS-CoV-2

**DOI:** 10.5811/westjem.2020.7.48725

**Published:** 2020-08-17

**Authors:** Rebecca Karb, Elizabeth Samuels, Rahul Vanjani, Catherine Trimbur, Anthony Napoli

**Affiliations:** * Alpert School of Medicine of Brown University, Department of Emergency Medicine, Providence, Rhode Island; † Alpert School of Medicine of Brown University, Department of Internal Medicine, Providence, Rhode Island

## Abstract

**Introduction:**

The unfolding COVID-19 pandemic has predictably followed the familiar contours of well established socioeconomic health inequities, exposing and often amplifying preexisting disparities. People living in homeless shelters are at higher risk of infection with severe acute respiratory syndrome coronavirus 2 (SARS-CoV-2) compared to the general population. The purpose of this study was to identify shelter characteristics that may be associated with higher transmission of severe acute respiratory syndrome coronavirus 2 (SARS-CoV-2).

**Methods:**

We conducted a cross-sectional assessment of five congregate shelters in Rhode Island. Shelter residents 18 years old and older were tested for SARS-CoV-2 from April 19–April 24, 2020. At time of testing, we collected participant characteristics, symptomatology, and vital signs. Shelter characteristics and infection control strategies were collected through a structured phone questionnaire with shelter administrators.

**Results:**

A total of 299 shelter residents (99%, 299/302) participated. Thirty-five (11.7%) tested positive for SARS-CoV-2. Shelter-level prevalence ranged from zero to 35%. Symptom prevalence did not vary by test result. Shelters with positive cases of SARS-CoV-2 were in more densely populated areas, had more transient resident populations, and instituted fewer physical distancing practices compared to shelters with no cases.

**Conclusion:**

SARS-CoV-2 prevalence varies with shelter characteristics but not individual symptoms. Policies that promote resident stability and physical distancing may help reduce SARS-CoV-2 transmission. Symptom screening alone is insufficient to prevent SARS-CoV-2 transmission. Frequent universal testing and congregate housing alternatives that promote stability may help reduce spread of infection.

## INTRODUCTION

People living in congregate homeless shelters are at higher risk of infection with severe acute respiratory syndrome coronavirus 2 (SARS-CoV-2) compared to the general population.^1^–^3^ Moreover, this population has a higher prevalence of baseline comorbidities that increase the risk of severe disease and mortality from SARS-CoV-2.^4^–^7^ While high rates of asymptomatic SARS-CoV-2 have been observed in homeless shelters, little is known about shelter-level risk factors and successful mitigation strategies. Many shelters have worked to comply with the US Centers for Disease Control and Prevention (CDC) recommendations to control transmission (eg, daily symptom screening and temperature checks).^3^ However, these mitigation strategies can be difficult and costly to implement and have unclear benefits. To date, no study has examined the association of shelter characteristics with SARS-CoV-2 outbreaks. In this analysis, we describe the varying prevalence of SARS-CoV-2 infection in five congregate homeless shelters in Rhode Island as well as varying shelter characteristics and infection control practices.

## METHODS

We conducted a cross-sectional assessment of congregate shelter residents 18 years of age and older staying in five shelters in Rhode Island, from April 19–April 24, 2020. Testing occurred during the peak of new case identification in Rhode Island. All residents of each shelter were offered testing. At the time of testing, we measured temporal temperature and pulse oximetry and collected information on demographic characteristics, comorbidities (hypertension, diabetes, heart disease, immunosuppression), and viral symptoms. Testing was done at Shelter 5 prior to initiation of temperature and oxygen documentation. Shelter characteristics and infection control practices were assessed by structured telephone interview with shelter administrators. Of note, shelter residents testing positive for SARS-CoV-2 in Rhode Island were being isolated in a hotel with support coordinated by the Rhode Island Department of Health (RIDOH). This screening was performed in collaboration with RIDOH to identify and isolate positive shelter residents.

We collected and managed data using REDCap (Vanderbilt, Nashville, TN). Nasopharyngeal swabbing was done by emergency physicians with training in appropriate nasopharyngeal swab technique. Tests were run on one of three available polymerase chain reaction (PCR) assays: Roche (specificity 99.8%, sensitivity 100%, Basel, Switzerland); Cepheid (specificity 99.2%, sensitivity 95.5%, Sunnyvale, CA); and Abbott (specificity 100%, sensitivity 93%, Chicago, IL).

We used descriptive statistics to summarize participant and shelter characteristics. We compared the proportion of positive SARS-CoV-2 tests among shelters, demographic groups, medical comorbidities, and symptomatology using t-tests and Fisher’s exact tests using STATA (Statacorp, College Station, TX). The analysis was deemed exempt by the RIDOH Institutional Review Board.

## RESULTS

Among 302 shelter residents across five shelters, 299 (99.0%) were tested for SARS-CoV-2; one person declined testing, and two declined to have their results included in the analysis. The overall case prevalence across all shelters was 11.7%. Approximately half of shelter residents were White (53%), about one quarter were Latinx (23%), and most were 40–64 years of age (61%, mean age 47.9 years of age) (Tables 1 and 2). More than a third reporting having asthma, chronic obstructive pulmonary disease, hypertension, diabetes, or heart disease (38%), with hypertension being the most prevalent comorbidity (23%) (Table 2). Demographic and shelter characteristics are shown in Table 1.

**Population Health Research Capsule**

What do we already know about this issue?

People living in congregate homeless shelters are at higher risk of infection with severe acute respiratory syndrome coronavirus 2 (SARS-CoV-2).

What was the research question?

What are shelter-level risk factors and successful mitigation strategies that impact the spread of SARS-CoV-2?

What was the major finding of the study?

Resident stability and physical distancing measures may reduce SARS-CoV-2 transmission in congregate settings.

How does this improve population health?

Symptom screening is insufficient to prevent spread in congregate shelters. Universal testing and stable housing alternatives could reduce risk for this population.

SARS-CoV-2 prevalence in Shelters 1 and 4 was 21.6% and 35.3%, respectively, while all other shelters had no cases (Table 1). There were no differences in age, gender, or race between people testing positive and negative for SARS-CoV-2 (Table 2). Only 20% of people testing positive (7/35) reported any symptoms; none had fever or hypoxia. There were no differences in the presence of symptoms between people testing positive and negative for SARS-CoV-2 (20.0% vs 14.0%, p = 0.34). People testing positive for SARS-CoV-2 had lower prevalence of comorbidities compared to people testing negative (20% SARS-CoV-2 positive vs 40% SARS-CoV-2 negative, p = 0.02). Among participants with negative tests, 70.1% (185/264) had spent more than two weeks at their shelter, compared to 42.9% (15/35) of participants with positive tests (p<0.001).

Regarding infection control practices, all five shelters required masks, performed daily temperature checks of clients and staff, provided onsite meals, and were open 24 hours (Table 1). Three shelters had stopped accepting new residents for at least two weeks prior to the study and had zero cases at time of testing. The shelter with the highest case positivity rate has several distinct characteristics compared to the other shelters (Table 1). The neighborhood of this shelter had higher census-tract population density compared to the neighborhoods of the other shelters. The resident population was also found to be more transient than that of other shelters, with only 58% (39/63) reporting staying at the shelter for more than two weeks. This low-threshold shelter has continued to keep its doors open to new residents throughout the pandemic, and given its limited capacity the shelter was unable to arrange sleeping areas at least six feet apart.

## DISCUSSION

The range of asymptomatic prevalence of SARS-CoV-2 found in different shelters builds on growing data from other cities and has important policy implications. Only one in five people with positive tests were symptomatic, which was not significantly different from those testing negative. Following initial CDC guidance, shelters have relied primarily on symptom screening to control spread of SARS-CoV-2. As this and other recent data have demonstrated, asymptomatic and pre-symptomatic transmission may be the predominant modes of SARS-CoV-2 spread in congregate settings, and thus symptom-guided identification and temperature screening are insufficient strategies to prevent SARS-CoV-2. Sheltering in place, wearing masks, and physical distancing may be at least partially effective in shelters with lower occupancy and a more stable resident population. However, low-threshold shelters provide an important safety net for many people experiencing homelessness, and shelters cannot be closed without readily available supportive housing.

Shelter characteristics and practices may play an important role in transmission and have not been adequately studied. This study found that shelters with more transient residents and operating at near-full capacity had higher prevalence rates. Shelters that limited the influx of new residents were able to prevent outbreaks; however, this practice comes at the cost of limiting access to individuals and families experiencing homelessness. The shelter with the highest prevalence of the virus was located in the most densely populated neighborhood. This finding underscores the potential importance of the surrounding neighborhood and indicates that future studies should examine area characteristics such as land-use mix and access to bus or train services.

To maintain low-threshold shelter services, use of frequent universal testing regardless of symptoms and ability to isolate people testing positive will be necessary for reducing SARS-CoV-2 transmission among people experiencing homelessness. Housing stability has been previously shown to improve health outcomes among people experiencing homelessness,^8^ and the importance of stable housing is readily apparent during the COVID-19 pandemic. Housing alternatives to large congregate shelters can be used to reduce transmission. This includes expansion of small, non-congregate shelter capacity as well as permanent supportive housing, which allows for more resident stability and improved physical isolation capabilities.

## LIMITATIONS

Although this study is the first to assess shelter-level characteristics, it was limited by the cross-sectional design as well as the small number of shelters. First, at the time of our study, many shelter residents who had tested positive were already housed in a local hotel, which likely led to an underestimate of true prevalence of SARS-CoV-2 among people experiencing homelessness. Second, testing done at the shelters with more transient residents only reflects the residents present on the night of testing, not the entire group that intermittently uses shelter services. Those shelters were more likely to have residents test positive; thus, an inability to assess the full complement of those shelters’ residents likely dilutes the overall prevalence of positivity when all shelters are examined in aggregate. Third, shelter staff are also a potential risk to residents, particularly if they work at multiple shelters/organizations or have other personal exposures. Our analysis does not account for potential risk posed by staff. Fourth, PCR tests used may have a 20–30% false negative rate and are only adequate during viral shedding.^9^ Furthermore, tests were conducted in three separate labs using different PCR assays with varying sensitivities/specificities. This may have impacted uniformity of test results. Lastly, since this was a cross-sectional analysis we were not able to determine whether the asymptomatic positive cases were actually presymptomatic.

## CONCLUSION

A growing body of literature has demonstrated that asymptomatic and presymptomatic spread of SARS-CoV-2 may be significant.^10^,^11^ The results of this study further underscore that symptom screening and temperature monitoring are insufficient means to mitigate transmission of SARS-CoV-2 in homeless shelters and other congregate settings. Shelter characteristics such as population density, the capacity to maintain population stability, and the ability and resources to implement preventative practices such as physical distancing, may be partially effective in mitigating disease spread. In order to prevent SARS-CoV-2 transmission while continuing to provide essential, accessible services to people experiencing homelessness, there is a need for frequent universal testing, infection control support at homeless shelters, and expanded availability of permanent housing.

## Figures and Tables

**Table 1:** SARS-CoV-2 prevalence, participant and shelter characteristics, and infection control practices, by homeless shelter.

	All	Shelter				

		1	2	3	4	5
Number Tested	299	51	89	48	68	43
SARS-CoV-2 +, n (%)	35 (11.7)	11 (21.6)	0	0	24 (35.3)	0
Participants Characteristics						
Age, mean (range)	47.9 (18–85)	43.4 (18–67)	48.5 (20–72)	47.8 (25–76)	46.7 (19–69)	53.7 (30–85)
Female, n (%)	59 (20)	18 (35)	0	32 (67)	9 (13)	0
Race, n (%)						
Black	59 (20)	11 (22)	17 (19)	5 (10)	17 (25)	9 (21)
White	160 (53)	18 (36)	50 (56)	35 (73)	36 (53)	21 (49)
American Indian/Alaska Native	10 (3)	1 (2)	3 (3)	3 (6)	3 (4)	0
Other/Unknown	70 (23)	21 (40)	19 (21)	5 (10)	12 (18)	13 (30)
Latino/a/x, n (%)						
Latino/a/x	68 (23)	14 (27)	15 (17)	11 (23)	12 (18)	16 (37)
Non-Latino/a/x	213 (71)	29 (57)	66 (74)	37 (77)	54 (79)	27 (63)
Other/Unknown	18 (6)	8 (15)	8 (9)	0	2 (3	0
Any comorbidities, n (%)	113 (38)	7 (13)	37 (42)	30 (63)	23 (34)	16 (37)
Any symptoms, n (%)	44 (15)	4 (8)	7 (8)	9 (19)	19 (28)	5 (12)
Shelter characteristics						
Census tract population density (number people per square mile)		10,852	2,753	10,852	21,645	2,362
% of beds filled (previous night)		100	88	90	97	100
% of population at shelter >14 days		*	82	96	58	98
Infection control practices						
Staff and residents wear masks		Yes	Yes	Yes	Yes	Yes
Daily temperature checks		Yes	Yes	Yes	Yes	Yes
Daily symptom screenings		Daily	2× Daily	Daily	Daily	2× daily
Onsite meals offered		Yes	Yes	Yes	Yes	Yes
Sleeping spaces 6 feet apart		Yes	No	Yes	No	Yes
Open 24 hours		Yes	Yes	Yes	Yes	Yes
Daily education/updates		No	Yes	No	No	No
New residents allowed		Yes	No	No	Yes	No

* Data type not collected at this shelter.

*SARS-CoV-2*, severe acute respiratory syndrome coronavirus 2.

**Table 2:** Demographic and clinical characteristics of participants, by SARS-CoV-2 result.

	All (N=299)	SARS-CoV-2 test		P-value

		Positive (N=35)	Negative (N=264)	
Shelter, n (%)				
1	52 (17)	11 (31)	40 (15)	< 0.001
2	89 (30)	0 (0)	89 (34)	
3	48 (16)	0 (0)	48 (18)	
4	68 (23)	24 (69)	44 (17)	
5	43 (14)	0 (0)	43 (16)	
Demographics				
Age, n (%)				
18–39	89 (30)	9 (25)	80 (31)	0.83
40–64	184 (61)	23 (66)	161 (61)	
>65	26 (9)	3 (9)	23 (8)	
Gender, n (%)				
Female	59 (20)	9 (26)	50 (19)	0.77
Male	238 (80)	26 (74)	212 (80)	
Trans/other	2 (1)	0 ()	2 (1)	
Race, n (%)				
Black	59 (20)	7 (20)	52 (20)	0.88
White	160 (53)	17 (49)	142 (54)	
Al/Alaska Native	10 (3)	1 (3)	9 (3)	
Other/Unknown	70 (23)	10 (29)	60 (23)	
Latino/a/x, n (%)				
Latino/a/x	68 (23)	6 (17)	62 (23)	0.001
Non-Latino/a/x	213 (71)	22 (63)	191 (73)	
Other/Unknown	18 (6)	7 (20)	11 (4)	
Transiency, n (%)				
>14 days at current shelter	200 (67)	15 (43)	185 (70)	< 0.001
Slept elsewhere	48 (16)	6 (17)	42 (16)	
Unknown	51 (17)	14 (40)	37 (14)	
Clinical				
Comorbidities, n (%)				
Any comorbidity	112 (38)	7 (20)	105 (40)	0.02
Asthma/COPD	52 (17)	1 (3)	51 (29)	
Hypertension	68 (23)	4 (11)	64 (24)	
Diabetes	32 (11)	2 (6)	30 (11)	
Heart disease	23 (8)	2 (6)	21 (8)	
Temperature, mean (SD)	97.1 (0.05)	96.8 (0.86)	97.2 (0.86)	0.06
Oxygen saturation, mean (SD)	96.7 (0.12)	97 (0.39)	96.7 (0.13)	0.59
Symptoms, n (%)				
Any symptoms	44 (14.7)	7 (20)	37 (14)	0.34
Fever	5 (2)	1 (3)	4 (2)	
Cough	15 (5)	2 (6)	13 (5)	
Shortness of breath	11 (4)	0 (0)	11 (4)	
Body aches	5 (2)	2 (6)	3 (1)	
Nausea, vomiting, or diarrhea	15 (5)	2 (6)	13 (5)	
Loss of smell or taste	9 (3)	2 (6)	7 (3)	

*SARS-CoV-2*, severe acute respiratory syndrome coronavirus 2; *COPD*, chronic obstructive pulmonary diseas; *SD*, standard deviation.
